# Chronic Periaortitis (Retroperitoneal Fibrosis) Concurrent with Recurrent Cutaneous Eosinophilic Vasculitis

**DOI:** 10.1155/2011/548634

**Published:** 2011-09-08

**Authors:** Despoina Kiorpelidou, Georgios Gaitanis, Aikaterini Zioga, Athina C. Tsili, Ioannis D. Bassukas

**Affiliations:** ^1^Department of Skin and Venereal Diseases, School of Medicine, University of Ioannina and University Hospital of Ioannina, 45110 Ioannina, Greece; ^2^Department of Pathology, School of Medicine, University of Ioannina and University Hospital of Ioannina, 45110 Ioannina, Greece; ^3^Department of Clinical Radiology, School of Medicine, University of Ioannina and University Hospital of Ioannina, 45110 Ioannina, Greece

## Abstract

Chronic periaortitis (CP) is usually accompanied by at least mild manifestations of systemic autoimmunity; however, skin manifestations are rare. Here, we report an 82-year-old woman presenting with a pruritic annular eosinophilic dermatosis that led to the diagnosis of recurrent cutaneous eosinophilic vasculitis (RCEV) coexisting with a latent CP. The present paper is reminder that a CP should be included as a potential differential diagnosis in the elaboration of patients with cutaneous vasculitis that is suspicious of underlying autoimmunity.

## 1. Introduction

Chronic periaortitis (CP) is an umbrella term used to describe a group of nosologically allied conditions that include idiopathic retroperitoneal fibrosis (Ormond's disease), inflammatory abdominal aortic aneurysm, and perianeurysmal retroperitoneal fibrosis [1, 2]. Most cases are idiopathic; however, cases of secondary CP to different triggering events (certain medications, infections, malignancies, and traumas) have been documented in the literature (reviewed in [2]). Common clinical feature of all these conditions is the development of an inflammatory fibrotic tissue mass in the retroperitoneal space that originates around the abdominal aorta and grows to entrap neighboring abdominal organs, particularly the ureters. The onset of CP is in most cases both insipient and of nonspecific nature. Localized symptoms connected to the growing-infiltrating retroperitoneal mass, like a dull abdominal, flank or back pain, or colic-like pain indicative of ureter entrapping, are the commonest presenting signs of CP. CP is usually accompanied by at least mild symptoms and signs of systemic autoimmunity [1]. In many cases, unspecific manifestations of a systemic disease (fatigue, anorexia, or low-grade fever) may mark disease onset. If diagnosed timely, CP can be effectively treated with steroids: however, insidious disease onset may result in significant delay in diagnosis with the consequences of ureteral obstruction and development of end-stage renal failure [2]. Skin manifestations are a rather exceptional event in all clinical forms of CP [2, 3]. Here, we report on a patient with a pruritic annular eosinophilic dermatosis as the heralding presentation of CP.

## 2. Case Report

**Figure 1:**
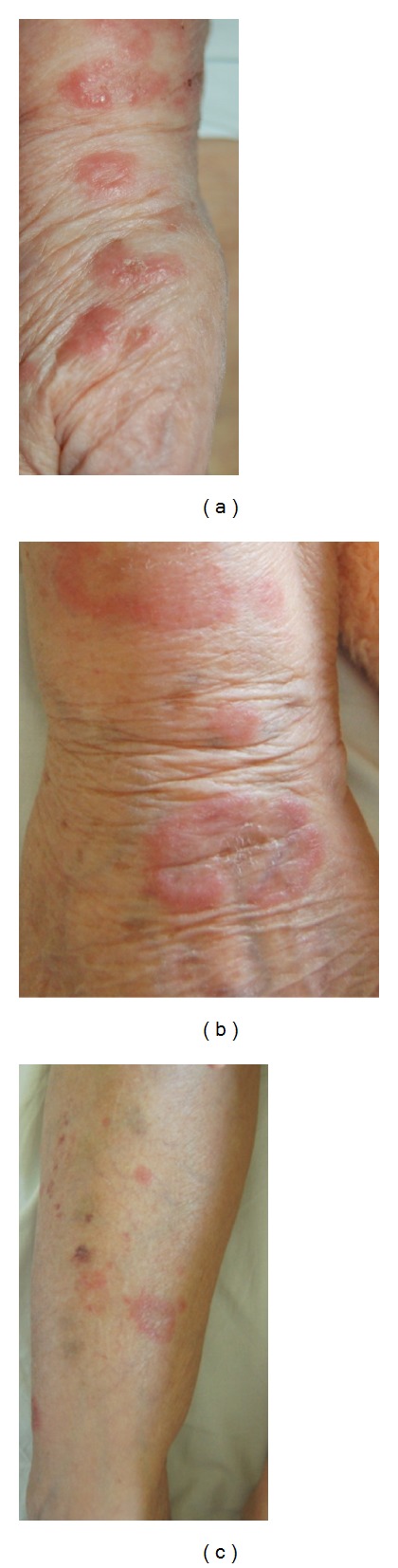
Chronic periaortitis presenting as recurrent cutaneous eosinophilic vasculitis (RCEV). (a, b) Urticarial, partly annular skin lesions of dorsal and medial aspects of right wrist-hand region. (c) Urticarial-erythematous skin lesions of the right leg; note signs of blood extravasations in healed lesions.

An 82-year-old woman presented with a two-month history of fatigue, anorexia, and pruritic skin lesions nonresponding to oral antihistamines. On admission, physical examination was unremarkable except for multiple polycyclic-annular, erythematous papules, and plaques mainly affecting her extremities, some with signs of central clearing and scales (Figures 1(a)–1(c)). Many lesions initially simulated wheals, yet they grew slowly over 1-2 weeks to fade subsequently leaving behind grayish-colored maculas (Figure 1(c)). Her medical history included coronary heart disease and hyperuricaemia on furosemide, metoprolol, isosorbide mononitrate, captopril, aspirin, and allopurinol at the time of admission. No new medications were instituted over the last year, even on per needed basis.

Laboratory tests revealed elevated erythrocyte sedimentation rate and increased C-reactive protein. Rest of laboratory evaluation was within the normal range or negative (Table 1).

**Figure 2:**
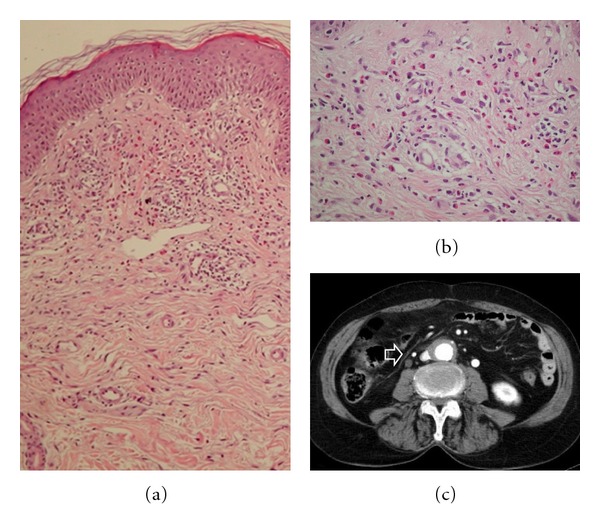
Chronic periaortitis presenting as recurrent cutaneous eosinophilic vasculitis (RCEV). (a) Skin biopsy showing under a focally slight spongiotic epidermis edema and moderately dense perivascular and interstitial inflammatory cell infiltrates confined to the upper dermis (hematoxylin & eosin; ×40). (b) Histopathology of skin lesion (detail) showing the inflammatory infiltrate consisting of lymphocytes, monocytes, and plentiful eosinophils. Altered vessels with endothelial swelling, intraluminal fibrin, a few eosinophils within vessel walls, and eosinophilic dust around them were featured. Leukocytoclasis or overt vascular necrosis was not seen (hematoxylin & eosin; ×160). (c) Contrast-enhanced CT scan (portal phase) revealed a mantle of a soft-tissue mass enveloping the aorta, consistent with chronic periaortitis (retroperitoneal fibrosis). Typically, the aorta is encased but not displaced by this process. Arrow: right hydroureter.

A lesional skin biopsy showed a moderately dense perivascular and interstitial inflammatory cell infiltrate in the upper dermis (Figure 2(a)), consisting of lymphocytes, monocytes, and plentiful eosinophils. Endothelial swelling, intraluminal fibrin, eosinophils within vessel walls and eosinophilic dust around them were featured, consistent with the diagnosis “eosinophilic vasculitis” (Figure 2(b)). Flame figures were not seen. Direct immunofluorescence studies were negative. Based on the clinical-pathologic correlation analysis and the differential diagnosis considerations (discussed in the next paragraph), a “recurrent cutaneous eosinophilic vasculitis” (RCEV) was diagnosed.

A series of conditions was included in the differential diagnosis of the present case. Because of the presence of annular lesions, some of them also with a “collaret-like” scale, an erythema annulare centrifugum (EAC) was initially considered in the differential diagnosis of the skin lesions of this patient. However, in the present case, the lesions are much infiltrated, oedematous, and quite numerous to be diagnosed as EAC, evolve too quickly for EAC, and also heal with signs of vasculitis. At tissue level, EAC is usually characterized by the presence of a moderately dense mixed cells infiltrate around vessels of the superficial vascular plexus of the dermis. Also eosinophils, sometimes plentiful, may be present. However, in contrast to the present case in EAC the infiltrate is typically well demarcated (“coat sleeve”) and morphologic findings of vasculitis fail. Wells' syndrome and hypereosinophilic syndrome were excluded in the present case because of the histopathological findings of skin inflammation confined to the perivascular area of the upper dermis and lack of blood eosinophilia. Moreover, microscopic signs of massive eosinophil degranulation failed. Also the diagnosis of sarcoidosis, at first considered in the differential diagnosis of this recurrent annular eruption with findings of systemic inflammation, was subsequently disregarded: radiological (chest X-ray) and laboratory findings (normal ACE and calcium levels in serum) as well as the histopathological picture of skin lesions without any granulomatous inflammation sufficiently permit exclusion of sarcoidosis. Finally, fully developed lesions of “classic” urticarial vasculitis present histopathologically as leukocytoclastic cutaneous vasculitis, not seen in the present case on the occasion of two lesional skin biopsies. On the other hand, the presence of (eosinophilic) vasculitis sufficientlydistinguishes the skin lesions of this patient from persistent urticaria [4, 5]. In conclusion, based on clinical, laboratory and biopsy findings, Wells' syndrome, hypereosinophilic syndrome, erythema annulare centrifugum (EAC), sarcoidosis and urticarial vasculitis were all excluded and the differential diagnosis of the eruption of this patient was narrowed down to “recurrent cutaneous eosinophilic vasculitis” (RCEV).

A subsequent abdominal computed tomography, prompted by persistently elevated ESR, revealed the presence of a soft-tissue mass enveloping the aorta (Figure 2(c)), from the renal arteries down to the aortic bifurcation, that caused right mild hydronephrosis and dilatation of the ipsilateral ureter, findings consistent with CP (retroperitoneal fibrosis).

Oral methylprednisolone (0.5 mg/kg/d) in combination with colchicine (1 mg/d) was initiated. Over the next month, the patient had gradual resolution and she decided on her own to discontinue the treatment. One month later, the eruption relapsed; a second skin biopsy was taken in order to exclude early mycosis fungoides with RCVE findings as the first. Oral corticosteroids were restarted at the same initial dose and tapered slowly over a three-month period. At six-month followup, the patient was free of skin lesions and symptoms and remained at the 12-month follow-up visit.

## 3. Discussion

Skin manifestations heralding the presentation of CP are a rather exceptional event. To our knowledge, only one case has been adequately documented in the literature. In that case, a recurring cutaneous vasculitis, probably of erythema elevatum and diutinum type, was diagnosed, that preceded the development of an advanced multifocal fibrosclerosis by many years [3]. The isolated report of a case of CP development in a patient with vitiligo [6] further supports this observation. Skin is rich in connective tissue and a common target organ in the course of diverse autoimmunity disorders. The pathophysiological mechanisms that underlie sparing of the skin in CP can only be addressed in future focused studies.

Different vasculitis syndromes belong to the most frequently described comorbidities of CP. Arteritis Takayasu [7–10], Henoch-Schönlein purpura [11, 12], polyarteritis nodosa [13], malignant atrophic papulosis [14], and livedo reticularis [15] all have been described sufficiently often to be significant. RCEV is a rare, relatively recently identified vasculitis entity, which in many cases has been described in association with connective tissue diseases (CTD) [16]. Most patients with RCEV present clinically with purpuric papules, pruritic nonblanching erythematous papules, or urticarial plaques; however, cases with pruritic annular eruptions have been also described [17]. RCEV is distinguished from persistent urticaria by the presence of vasculitis in histology [18]. Vasculitis of the small retroperitoneal vessels [1] and infiltration by eosinophils [19] are frequent characteristic histopathologic findings in the fibrotic lesions of CP. The present description of RCEV in a patient with CP raises the possibility that a milieu of eosinophil activation may be directly implicated in the pathogenesis of the small vessel vasculitis of CP. A recently published mouse model described an IgE-mediated Arthus reaction as a feasible mechanism leading to eosinophilic vasculitis [20]. Future studies should clarify whether a similar hypersensitivity reaction contributes to the development of the vasculitis of CP too. Nevertheless, given the established connection of RCEV to autoimmunity [16], the present description of a CP presenting as RCEV is an additional argument in favor of the proposed autoimmune nature of CP [1]. 

Like RCEV [16], CP may also present in association with a variety of CTD [1, 2]. This raises the possibility of coincidence of the two entities in our patient as manifestations of a common underlying CTD. Annular skin lesions are frequently found in the course of different autoimmune conditions, including Sjögren's syndrome (SS) [21], which has been also reported in association with CP [22]. Initially SS was suspected in this elderly patient with the pruritic annular eruption; yet, criteria for SS diagnosis were not fulfilled. Similarly, no evidence was found for a systemic angiitis, particularly Churg-Strauss syndrome or (hypocomplementaemic) urticarial vasculitis, conditions that could underlie both RCEV and CP [2, 16].

In conclusion, the present paper is a reminder that a latent CP should be included in the elaboration of patients with cutaneous vasculitis that is suspicious of underlying autoimmunity. CP usually lacks early signs and in most cases a symptomatic advanced-stage disease is diagnosed. Yet, CP responses promptly to corticosteroids [2] and a timely onset of treatment should prevent the development of severe complications, such as end-stage renal failure.

## Figures and Tables

**Table 1:** Compilation of the results of laboratory investigations at presentation.

*Investigations with pathological results*
(i) Erythrocyte sedimentation rate: 107 mm/h
(ii) C-reactive protein (CRP): 30.1 mg/L (normal: <6 mg/L)
(iii) High sensitivity CRP: 27.0 mg/L (normal: <5 mg/L)
(iv) Abdomen computed tomography (finding: chronic
periaortitis)

*Investigations with normal or negative results*
(i) Full blood count (including eosinophil count), lymphocyte
subpopulation by flow cytometry of peripheral blood,
hemoglobin, serum ferritin
(ii) Serum: electrolytes (including Ca and PO_4_), fasting blood
sugar, fasten lipids, urea, creatinine, uric acid, angiotensin
converting enzyme (ACE)
(iii) Liver and thyroid function tests
(iv) Blood coagulation parameters, serum D-dimers
(v) Urine chemistry and sediment
(vi) Antinuclear antibody (ANA)^1^, extractable nuclear antigens
(ENA), double-stranded DNA, antismooth muscle antibodies
(ASMA), antimitochondrial antibodies (AMA), ANCA (-P, -C,
-MPO and -PR3), anti-Ro/SSA, anti-La/SSB, rheumatoid
factor,anticardiolipin antibodies (IgG and IgM)
(vii) Complement levels; serum proteins electrophoretogram;
serum immunoglobulins by immune electrophoretogram
(including IgA and IgE)
(viii) ASTO, RPR, serology for *Borrelia burgdorferi*,
*Echinococcus*, *Toxocara canis*, Hepatitis B and C viruses,
Epstein-Barr virus and HIV
(ix) Serological tumor markers (*α*-Fetoprotein (*α*-FP),
carcinoembryonic antibody (CEA), Ca19.9, Ca15.3, Ca125)
(x) Tuberculin skin test (TST = 2 mm)
(xi) Schirmer's test
(xii) Chest imaging (X-ray and computed tomography)^2^

^1^Borderline positive (1 : 80, speckled pattern) at first examination; repeatedly negative (<1 : 80) on subsequent testing.

^2^Except for signs of chronic heart failure.
